# Study of Evolution of Microbiological Properties in Sewage Sludge-Amended Soils: A Pilot Experience

**DOI:** 10.3390/ijerph17186696

**Published:** 2020-09-14

**Authors:** Natividad Miguel, Judith Sarasa, Andrea López, Jairo Gómez, Rosa Mosteo, María P. Ormad

**Affiliations:** 1 “Agua y Salud Ambiental” Research Group, Department of Chemical Engineering and Environmental Technologies, Instituto de Investigación en Ciencias Ambientales (IUCA), University of Zaragoza, 12 Pedro Cerbuna Street, 50009 Zaragoza, Spain; jsarasa@unizar.es (J.S.); mosteo@unizar.es (R.M.); mpormad@unizar.es (M.P.O.); 2 Navarra de Infraestructuras Locales S.A., 31008 Pamplona, Spain; alopez@nilsa.com (A.L.); jgomez@nilsa.com (J.G.)

**Keywords:** sewage sludge, soil properties, microbiological pollution, clayey soil, sandy soil, pathogenic bacteria

## Abstract

Large amounts of sewage sludge are generated in urban wastewater treatment plants and used as fertilizer in agriculture due to its characteristics. They can contain contaminants such as heavy metals and pathogenic microorganisms. The objective of this research work is to study, in real conditions, the evolution of microbial concentration in agricultural soils fertilized by biologically treated sewage sludge. The sludge (6.25 tons Ha^−1^) was applied in two agricultural soils with different textures and crops. A microbiological (total coliforms, *Escherichia coli*, *Staphylococcus aureus*, *Enterococcus* sp., *Pseudomonas* sp., *Salmonella* sp. and total mesophylls) and physical-chemical characterization of the sludge, soils and irrigation water were carried out. The evolution of these parameters during sowing, growth and harvesting of crops was studied. Initially, sewage sludge had a higher concentration of microorganisms than soils. Irrigation water also contained microorganisms, fewer than sewage sludge amendment but not negligible. After amendment, there were no differences in the microbiological evolution in the two types of soil. In general, bacterial concentrations after crop harvest were lower than bacterial concentrations detected before sewage sludge amendment. Consequently, the application of sludge from water treatment processes did not worsen the microbiological quality of agricultural soil in this study at real conditions.

## 1. Introduction

Sewage sludge is waste generated in various stages of urban wastewater treatment. It is a mixture of water and solids separated from wastewater by means of natural or artificial processes. The greatest volumes of sludge are generated in primary and secondary decanters. As sludge is the main waste produced in wastewater treatment plants (WWTP), complementary treatments such as concentration, dewatering, aerobic or anaerobic digestion, etc. must be carried out. The selection of adequate treatments depends on the final destination of the sludge and they have to guarantee environmentally safe management. 

Among the various final destinations for sludge (land application, landfilling, incineration, ocean dumping and lagooning) [1], land application as fertilizer is the most advisable. On the one hand, sludge is rich in nutrients (N and P) and organic matter and, on the other hand, its use as fertilizer is a form of recycling, according to Directive 2008/98/EC of the European Parliament and of the Council of 19 November 2008, on waste and repealing certain Directives [2]. This directive establishes a hierarchy for waste prevention and management and prioritizes fertilizer use over other forms of recovery (e.g., energy recovery) and disposal.

In Spain, approximately 1,080,000 tons/year of sludge (as dried solids) were produced in 2010–2012 [3]. In 2010, the most widespread stabilization treatment carried out for sludge was anaerobic digestion (49%), followed by extended aeration (32%, most corresponding to WWTP of <5000 eq/inhab.) and aerobic digestion (8%). In 2012, 80% of generated sludge was used as fertilizer in agriculture, almost reaching the objective for the year 2020. The quantitative objective for 2020 is a minimum of 85% of material valorization (in agricultural land or others). Several regions in Spain reached this objective in 2012. La Rioja and Navarra are the two regions that applied 100% of generated sludge to agricultural land. The use of sludge in agriculture is regulated in Spain by Royal Decree 1310/1990 [4], which transposes the Directive 86/278/CEE of 12 June 1986 on the protection of the environment, and in particular of the soil, when sewage sludge is used in agriculture [5]. This legislation establishes limits on the content of seven heavy metals but not for other organic and inorganic pollutants or pathogens that can be found in sewage sludge [6,7]. It is known that several pathogenic microorganisms may be found in sludge derived from faecal material: bacteria (*Salmonella* spp., *Escherichia coli*, *Pseudomonas aeruginosa*, etc.), viruses, protozoa, helminths, etc. Being conscious of this fact, the current directive is under revision, and several member states have already implemented stricter limit values for heavy metals and set requirements for other contaminants such as pathogens [8]. Although there are no limit values for pathogens in Spanish legislation, facilities such as WWTPs, sludge treatment facilities and agents carrying out agricultural application must provide information about the concentration of *Salmonella* and *Escherichia coli* in these sludges, according to Orden AAA/1072/2013 relating to the use of sewage sludge in the agrarian sector [9].

Sludge for agricultural use is normally treated by means of mesophilic/thermophilic anaerobic or aerobic digestion, reducing pathogen levels but not eliminating them completely [10,11,12]. The recommended quality criteria of treated sludge take into account both the presence of pathogens and the sewage sludge’s attractiveness to vectors (e.g., rodents, flies, mosquitoes) [13,14,15]. The application of sludge must ensure the protection of human health and the environment; however, few studies have been done at real scale relating to the behavior, survival and evolution of pathogens in soils fertilized by sewage sludge. The reported results and conclusions found by other authors depend on a great number of variables: experimental conditions, climatology, period of analysis, type of soils and sludge, etc. Given the numerous environmental parameters influencing the survival of micro-organisms and the complexity of their interaction, it is not surprising that the results obtained by different workers do not always agree [16,17,18]. Some studies show that the numbers of microorganisms are normally 100–1000 times higher in sludge than in soils [17,19,20]. In general, it can be said that the evolution of microorganisms after land application depends essentially on the physical-chemical conditions of the soil and the availability of nutrients, the atmospheric conditions being less relevant [16,19]. The presence of pathogens in soils is influenced by soil moisture, aeration, soil texture, temperature, pH, ultraviolet (UV) radiation, etc. [16,17,18]. Soil biota is also involved [20,21,22,23,24]. The reduction of microorganisms is faster in soils fertilized by sludge than non-fertilized soils [23]. Pathogen survival in soils is related with the treatment or stabilization method, the time of storage of the sludge and the applied dosage [17,18]. For example, *Enterococcus* reduction relies on the aerobic or anaerobic treatment of the sludge [20,25] and *Escherichia coli* and *Enterococcus* reduction depends on temperature and soil texture soil and its characteristics [17,18]. It has been observed that the number of *Clostridium* spores increases and remains for months [26]. The growth of *Clostridium* in the environment may be due to the existence of anaerobic rooms [27].

This research work has been carried out in collaboration with N.I.L.S.A. (Navarra de Infraestructuras Locales S.A.), and its main objective is to study, under real conditions, the evolution of microbial concentration in agricultural land fertilized by urban wastewater sludge. This sludge was previously treated by aerobic digestion, and applied to sandy and clayey soils. Additionally, two species with different requirements of nutrients and water were cultivated: corn and sunflower. This study is focused on several Gram + and Gram – microorganisms (aerobic and aeroanaerobic): total coliforms, *Escherichia coli*, *Staphylococcus aureus*, *Enterococcus* sp., *Pseudomonas* sp., *Salmonella* sp. and total mesophilic bacteria. Characterization of the sludge and soils has been carried out at the different stages of plant cultivation, growing and harvest. The microbiological quality of the irrigation water has also been analyzed. The final objective is to determine the presence of pathogens during a complete vegetative cycle and establish the potential risk to human health and the environment.

## 2. Materials and Methods 

### 2.1. Sewage Sludge, Agricultural Soils and Irrigation Water

The sludge used in this study comes from an urban WWTP (capacity equivalent to 83.000 inhabitants) located in the Navarra region (Spain). This WWTP treats 490 m^3^ of sludge (coming from primary and secondary treatment) per week by aerobic digestion.

The soils used in this study, 4 Ha in size, are from agricultural land situated near the WWTP and within a distance of 1 km one from other. One soil has a clayey texture (clay content >30%) and the other has a sandy texture (clay content < 10%). The clayey soil has a particle size less than 0.002 mm, high compacity, water retention and thermal exchange capacity, and low permeability and aeration. On the other hand, the sandy soil has a particle size between 0.02 and 2.0 mm, low compacity, water retention and thermal exchange capacity, and high permeability and aeration [28].

The irrigation water comes from a channel located near the agricultural soils. Its origin is a river in the Ebro hydrographic basin. 

### 2.2. Crops

During this study, corn was sown in the clayey soil and sunflower in the sandy soil. The nutritional and irrigation needs of these crops are shown in Table 1 [29,30].

### 2.3. Application of Sewage Sludge to Agricultural Soils

Prior to the application of sewage sludge treated by aerobic digestion, the agricultural soils were loosened and aerated using a rototiller. A quantity of 25 tons of sewage sludge (according to the soil needs) were added to each soil (6.25 t Ha^−1^) in May 2018. This addition was made by surface distribution using an agricultural fertilizer spreader. Once the cultivation surface was covered, it was tilled in order to turn over the top centimeters of the surface soil with the sludge. Two days after the conditioning and preparation of the soils, the corresponding crops were sown with an agricultural planter.

### 2.4. Sampling 

The samples analyzed in this study were: (1) treated sewage sludge, previous to their application on soils (sludge samples, S_sludge_); (2) agricultural soils before being amended with sludge: clayey soil (clayey soil samples, S_0-clay_) and sandy soil (sandy soil samples, S_0-sand_); and soils amended with sewage sludge taken at four different times: (3) initially (S_i-clay_, S_i-sand_), (4) during the growth of crops, 3 weeks after amendment (S_3w-clay_, S_3w-sand_), (5) 5 weeks after amendment (S_5w-clay_, S_5w-sand_) and (6) at harvest time, after 42 weeks for corn and 24 weeks for sunflower (S_42w-clay_, S_24w-sand_). Moreover, samples of irrigation water used in the soils were collected (water samples, S_w_). 

Solid samples of soils and amended soils were taken following a standard method [31]. The soils were divided into squares of 324 m^2^ (124 squares per soil). A portion of soil was taken at a depth of 15 cm from each square. All the soil portions were homogenized. The quartering method was applied to the homogenized sample until a sample of 500 g was obtained for analysis. 

The sampling of sewage sludge and water followed standard methods ISO 5667–13:2011 and 5667–3:2018, respectively [32,33].

### 2.5. Analytical Methodology

A pre-treatment of solid samples (sludge and soils) was undertaken in order to analyze the microbiological and physico-chemical parameters. This pre-treatment was based on that described by Carter (1993) and consisted of taking 10 g of solid sample and adding 90 mL of distilled water, stirring the mixture at 3500 rpm for 25 min. The resulting aqueous samples and irrigation water samples were analyzed following the standard methods described below.

#### 2.5.1. Microbiological Parameters

The microbiological parameters analyzed in all the samples, the culture media used and the standard methods of analysis are shown in Table 2.

At the beginning of the microbiological analysis, the bacterial concentration in the samples was unknown. For this reason, serial dilutions were carried out in all the samples. In this way, the bacterial concentration was reliably determined. The serial dilutions were carried out dissolving 1 mL of sample in 9 mL of NaCl 0.9%. All the samples were analyzed using the plate count method. After sowing on the surface or using the membrane filtration method, the samples were subjected to the appropriate incubation period for each bacteria (time and temperature), resulting in plates with colored colonies that could be counted as colony-forming units (CFU). All the analyses were undertaken in triplicate. The microbiological concentration of solid samples (sludge, soils and amended soils) was given as CFU per gram of dry matter (measured as total solids, see Table 3) and the microbiological concentration of irrigation water was given as CFU per 100 mL.

#### 2.5.2. Physical and Chemical Parameters

The physical and chemical parameters analyzed in all the samples, the equipment used and the standard methods of analysis are shown in Table 3. 

The suspended solids were only analyzed in the irrigation water samples. On the other hand, total solids, organic nitrogen and assimilable phosphorous and potassium, calcium, iron, magnesium and heavy metals were only analyzed in the solid samples. All the analyses were undertaken in triplicate. 

## 3. Results and Discussion

### 3.1. Initial Properties of Treated Sewage Sludge, Agricultural Soils and Irrigation Water

Table 4 shows the microorganism concentrations present in the treated sewage sludge, in the soils prior to amendment and in the irrigation water.

According to the results obtained in the solid samples, the highest bacterial concentration was found in the sewage sludge although concentrations of total coliforms, *Escherichia coli*, *Staphylococcus aureus* and total mesophylls were similar or only slightly higher in the sludge than in the soils. However, the sludge had a significantly higher concentration of *Enterococcus* sp. and *Pseudomonas* sp. than the soils. The predominant bacteria in the sewage sludge were total mesophylls, total coliforms and *Enterococcus* sp., the first two bacteria also being the predominant ones in both soils (10^5^–10^7^ CFU g^−1^). *Salmonella* sp. was not detected in any sample although this bacteria has commonly been found in sewage sludge in other studies [21,41,42,43].

The bacterial concentration detected in the irrigation water was between 10^1^ and 10^3^ CFU 100 mL^−1^, the total coliforms, *Pseudomonas* sp. and total mesophylls being the predominant bacteria. *Salmonella* sp. was not detected. Taking into account the crop water needs (~6.000–7.500 m^3^ Ha^−1^, corn-clayey soil; ~4.000–5.000 m^3^ Ha^−1^, sunflower-sandy soil) [29,30] and with the objective of comparing the contribution to the total bacteria by the sludge and irrigation water, it was estimated that during the whole period the total contribution to the bacteria concentration by the sludge was about 10^14^ CFU Ha^−1^ and by irrigation water about 10^11^ CFU Ha^−1^, in both soils. Therefore, the total bacterial contribution by sewage sludge amendment was greater than by irrigation water, although the latter was not negligible.

The initial physical-chemical properties of the treated sewage sludge, the agricultural soils prior to amendment and the irrigation water are shown in Table 5. 

According to the data obtained from the sludge and soils (prior to amendment), some properties were very similar: the pH, temperature and total solids. On the other hand, the soils had higher conductivity, calcium, iron and magnesium than sewage sludge. Both soils had neutral pH, with a certain degree of salinity, especially the sandy soil (>4000 μS cm^−1^), with a medium level of fertility according to the content of assimilable phosphorous and potassium and the low content of calcium and magnesium [44]. The only heavy metals detected, in similar concentrations in the sludge and soils, were nickel and zinc. The sewage sludge also contained copper. In general, the heavy metal concentrations detected in the sludge used in this study were similar to those reported in the literature [45,46,47]. The heavy metal concentrations detected did not exceed the present legislation criteria [4]. The other physical and chemical parameters show that the sludge had a greater organic matter and nutrient content than the agricultural soils, which indicates that sludge can be used as fertilizer due to its valuable organic matter and macroelement content (nitrogen and phosphorous) [48,49,50] necessary for the good development of the soil-plant ecosystem [51,52,53]. Both nitrogen and phosphorus are necessary for a range of compounds, the construction of cellular structures and enabling metabolic processes. Supplementation with these macroelements has a positive effect on the physiological state of plants and promotes the proper development of the root system [54].

The physico-chemical characteristics obtained for the irrigation water reflected typical values of the type of water used for this purpose [55].

### 3.2. Evolution of the Microbiological Quality of Amended Soils

**Figure 1:**
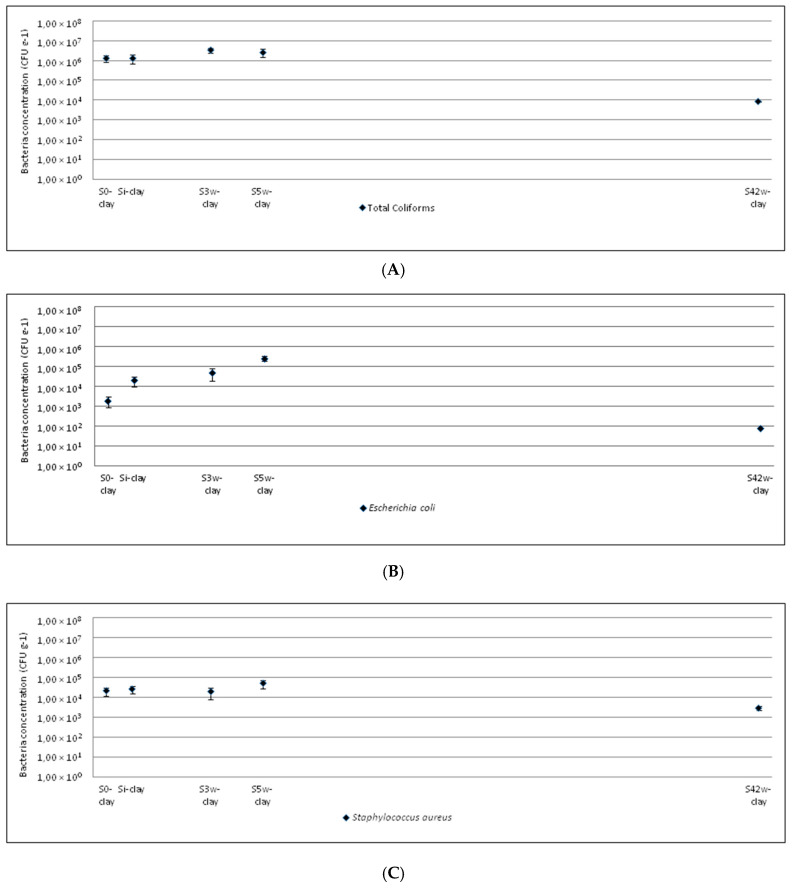

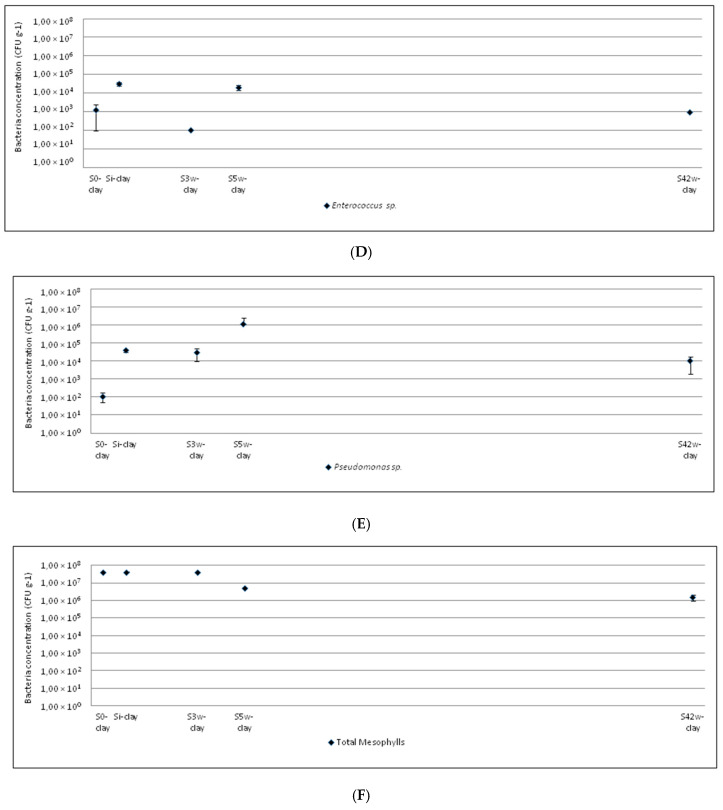
Evolution of microorganism concentrations in clayey soil amended with sewage sludge: (**A**) total coliforms; (**B**) *Escherichia coli;* (**C**) *Staphylococcus aureus*; (**D**) *Enterococcus* sp.; (**E**) *Pseudomonas* sp.; (**F**) total mesophylls.

**Figure 2:**
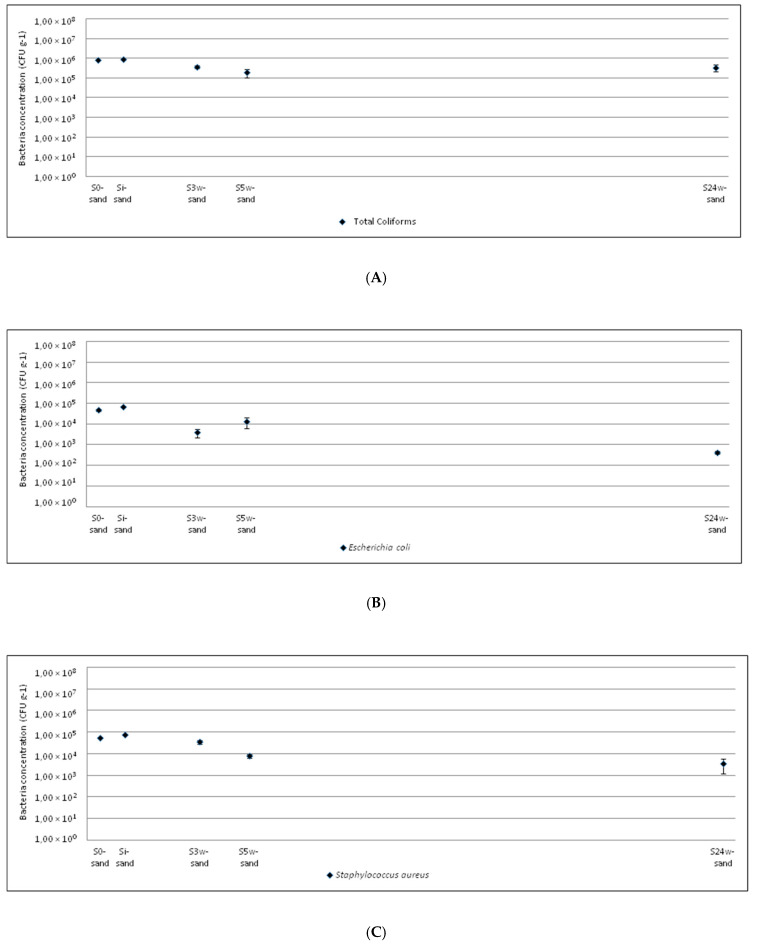

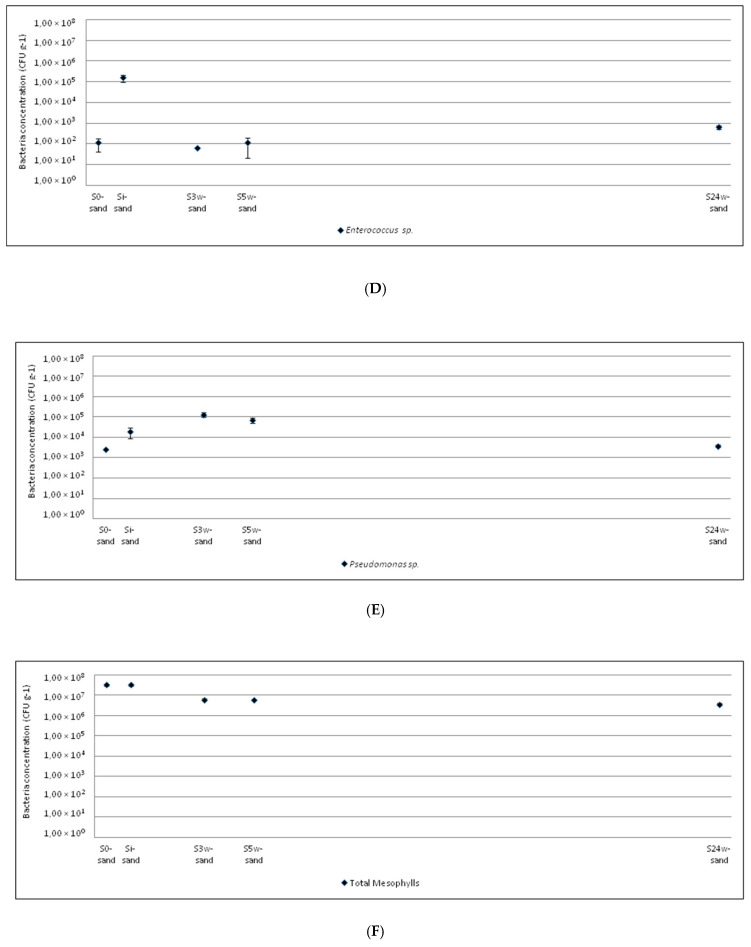
Evolution of microorganism concentrations in sandy soil amended with sewage sludge: (**A**) total coliforms; (**B**) *Escherichia coli*; (**C**) *Staphylococcus aureus*; (**D**) *Enterococcus* sp.; (**E**) *Pseudomonas* sp.; (**F**) total mesophylls.

The evolution of the microbiological quality of the clayey and sandy soils from amendment to crop harvest is shown in Figure 1 and Figure 2, respectively. 

In the case of total coliforms, the amendment with sewage sludge did not increase the bacterial concentration in soils with respect to the initial concentration. The variation in bacterial concentration was low during crop growth (S_3w_, S_5w_). At harvest, the bacterial concentration decreased about 10^3^ CFU g^−1^ in the clayey soil (S_42w-clay_). This decrease was not observed in the sandy soil (S_24w-sand_), probably because the growth time of sunflower (in the clayey soil) is considerably less than that of corn (in the sandy soil). This appears to contradict other studies that show the permanence of these bacteria in agricultural soils for long periods of time after the application of sewage sludge [22,56]. However, in some of these studies the quantity of sludge applied to agricultural soils was higher than in the present study. Moreover, the bacterial persistence also depends on other factors such as soil pH, organic matter, humidity, etc. [22,57] and the crops and their rooting systems can play big roles in shaping the microbial communities including their transport, survival, etc. [16].

The sewage sludge amendment produced an increase in *Escherichia coli* compared with its initial concentration in the clayey soil (S_i-clay_), but not in the sandy soil (S_i-sand_). During crop growth (S_3w_, S_5w_), the *Escherichia coli* concentration increased between 3–5 weeks after amendment. Finally, the bacterium concentration notably decreased to 10^1^–10^2^ CFU g^−1^ (S_42w-clay_, S_24w-sand_), lower than the initial concentrations in the agricultural soils (S_0-clay_, S_0-sand_).

*Staphylococcus aureus* had the same tendency in both agricultural soils. The amendment with sewage sludge (S_i-clay_, S_i-sand_) did not produce an increase in this bacterium in comparison with the initial concentration (S_0-clay_, S_0-sand_). During crop growth (S_3w_, S_5w_), the *Staphylococcus aureus* concentration slightly varied and at the crop harvest (S_42w-clay_, S_24w-sand_) it was lower than at the beginning (S_0-clay_, S_0-sand_). 

The increase in *Enterococcus* sp. following the amendment with sewage sludge is significant. This is consistent with the results of other studies [58]. The *Enterococcus* sp. concentration in both soils decreased 3 weeks after the amendment (S_3w-clay_, S_3w-sand_). After this, the concentration increased back up to the initial values prior to the amendment. This could be because *Enterococcus* sp. are intestinal bacteria and their survival in the environment is difficult until they are habituated to the edapho-climatic conditions. Factors such as soil humidity, temperature and the availability of nutrients can influence the pathogen reduction [19,20,24].

For *Pseudomonas* sp., the amendment with sludge (S_i-clay_, S_i-sand_) produced an increase in this bacterium concentration in the soils of 10^1^–10^2^ CFU g^−1^. During the crop growth in both soils, *Pseudomonas* sp. increased during 3–5 weeks after amendment (S_3w_, S_5w_). Finally, this concentration decreased to similar concentrations detected in the sandy soil prior to the amendment (S_0-sand_) whereas higher concentrations were detected in the clayey soil (S_0-clay_). *Pseudomonas* sp. are environmental bacteria and the variations in their concentration can be due to several factors. On the one hand, an increase in their concentration may be related to the contribution through the irrigation water for which there is a greater demand by corn (~6.000–7.500 m^3^ Ha^−1^, in the clayey soil) than by sunflower (~4.000–5.000 m^3^ Ha^−1^, in the sandy soil) [29,30]. According to the corn water needs, the contribution of *Pseudomonas* sp. by irrigation water (~10^11^ CFU Ha^−1^) is similar to that provided by the sewage sludge (~10^12^ CFU Ha^−1^) and slightly higher than the contribution due to the sunflower irrigation water (~10^10^ CFU Ha^−1^). On the other hand, both an increase and a decrease in the concentration may be due to environmental and specific factors pertaining to agricultural soils, such as temperature, ultraviolet radiation, soil humidity, soil pH, etc. [23].

Finally, the total mesophylls, which had the highest concentrations in all the samples, experienced very slight variations throughout the entire process. There was no significant variation either after the fertilization with sludge (S_i-clay_, S_i-sand_) or during the growth of both crops (S_3w_, S_5w_). At the time of the harvest of both crops (S_42w-clay_, S_24w-sand_), a concentration slightly lower than the initial one was found (S_0-clay_, S_0-sand_).

Again, *Salmonella* sp. was not detected in any case.

### 3.3. Evolution of the Physical-Chemical Quality of Amended Soils

The evolution of the physical-chemical parameters of the clayey and sandy soils during the amendment, growth and harvest of the crops is shown in Table 6 and Table 7, respectively.

The evolution of the physico-chemical parameters reveals no significant differences in either of the soils. The low variations in some analyzed parameters are due to the textures of both soils and the edapho-climatic conditions of the area.

It is important to note the contribution that the sewage sludge amendment produces in terms of organic matter and nitrogen concentration, as can be seen in Table 6 and Table 7. It produces an increase in organic matter (measured as total organic carbon) of 2.3 times the initial quantity in the clayey soil (S_0-clay_) and 2 times the initial quantity in the sandy soil (S_0-sand_). Furthermore, this causes an increase in nitrogen of 1.7 times the initial quantity in the clayey soil (S_0-clay_) and almost 2 times the initial quantity in the sandy soil (S_0-sand_). The soils under study have typical concentrations of organic matter (S_0_). The concentrations are low, in no case exceeding 1.4% of organic matter content in the sandy soil and 1.1% in the clayey soil. The desirable levels of organic matter for the satisfactory growth of crops are a minimum of 2% for clayey soils and 2.5% for sandy soils [59]. The amendment with sewage sludge achieves an increase in organic matter content up to desirable levels. The application, therefore, provides a beneficial contribution for the soils under study, as far as organic matter is concerned. Furthermore, this concentration remains practically constant during the crop growth. Regarding the nutritive elements necessary for the proper growth of crops (mainly nitrogen, phosphorous and potassium), the sewage sludge amendment produces suitable values to satisfy the nutritional needs of crops in both soils (24 kg N Ha^−1^, 11 kg P_2_O_5_ Ha^−1^ and 20 kg K_2_O Ha^−1^ for corn; 35 kg N Ha^−1^, 18 kg P_2_O_5_ Ha^−1^ and 35 kg K_2_O Ha^−1^ for sunflower) [44]. 

In addition, it has been demonstrated that after the pilot study the crops grew and were harvested with absolute normality.

## 4. Conclusions

The results of this research work show that the sewage sludge used in the study can be applied for the fertilization of agricultural soils given that it meets the quality criteria currently regulated in the legislation. The sewage sludge contains concentrations mainly between 10^5^ and 10^7^ CFU g^−1^ of total coliforms, *Escherichia coli*, *Staphylococcus aureus*, *Enterococcus* sp., *Pseudomonas* sp. and total mesophilic bacteria. It does not contain *Salmonella* sp. The agricultural soils used in this pilot experiment also contain potentially pathogenic bacteria, specifically concentrations between 10^2^ and 10^7^ CFU g^−1^ of the bacteria present in sludge. In most cases, the bacterial concentration of the sludge exceeded the bacterial concentration of the non-amended soils. The irrigation water also contained microbiological pollutants. Taking into account the water needs of the crops (corn and sunflower), the bacterial contribution by irrigation water was lower (although significant) than by sewage sludge except for *Pseudomonas* sp. which was similar for both (in the case of clayey soil).

Sewage sludge was applied to clayey and sandy agricultural soil in which corn and sunflower was planted, respectively. After the application of the sewage sludge, the bacterial concentrations under study experienced small variations over time but no significantly different trends were found in either soil. During the planting, growth and harvesting of crops, the bacterial concentration increased or decreased depending on the bacteria. In general, bacterial concentrations after the crop harvest were lower than those detected prior to the amendment with treated sewage sludge. Thus, the amendment with sewage sludge treated by aerobic digestion in this pilot experiment did not lead to a reduction in the microbiological quality of the soils under study. Only the *Pseudomonas* sp. concentration in the clayey soil was higher at the end than initially, probably due to the contribution of the irrigation water. Moreover, the amendment with sewage sludge produced an increase in the organic matter and nutrient contents which improve agricultural soils.

## Figures and Tables

**Table 1:** Nutritional and irrigation needs of crops.

Needs	Corn	Sunflower
Nitrogen	24.7–30.0 (kg t^−1^)	30.0–40.0 (kg t^−1^)
Phosphorous	10.2–12.3 (kg t^−1^)	15.0–20.0 (kg t^−1^)
Potassium	20.7–25.2 (kg t^−1^)	30.0–40.0(kg t^−1^)
Irrigation water	6000–7500 m^3^ Ha^−1^	4000–5000 m^3^ Ha^−1^

**Table 2:** Microbiological parameters, culture media and standard methods.

Bacterium	Culture Media	Standard Method	Reference
Total Coliforms	Chromogenic Coliform Agar (CCA)	ISO 9308–1	[34]
		9215B-C-D	[35]
*Escherichia coli*	Chromogenic Coliform Agar (CCA)	ISO 9308–1	[34]
	Glucuronic Agar tryptone and bile (TBX)	9215B-C-D 9222D	[35]
*Staphylococcus aureus*	Mannitol Agar	ISO 6888–1	[36]
	Nutritive Agar + NaCl (20%)	9215C	[35]
*Enterococcus* sp.	Slanetx and Bartley Agar	ISO 7899–2	[37]
		9215B-C-D	[35]
*Pseudomonas* sp.	Cetrimide Agar	UNE-EN ISO 16266	[38]
		9215C	[35]
*Salmonella* sp.	Xylose-Lysine-Desoxycholate (XLD) Agar Chromogenic Agar Salmonella Latex test	UNE-EN ISO 6579	[39]
Total Mesophylls	Nutritive Agar	9215B	[35]

**Table 3:** Physico-chemical parameters, equipment and standard methods.

Parameter	Equipment	Standard Method	Reference
pH	Multiparameter meter Orion Star A3295	4500H ^+^ -B	[35]
Temperature			
Conductivity	Conductimeter Hanna HI 9033	UNE-EN 27888–1994	[40]
Total Organic Carbon	Analyzer Shimadzu	5310B	[35]
Total Solids	Balance, heater	2540B	[35]
Suspended Solids	Balance, heater	2540D	[35]
Organic Nitrogen	Digester	4500-Norg	[35]
Assimilable Phosphorous	-	Olsen Method 4500-P	[35]
Assimilable Potassium	Atomic absorption spectrometer	3111	[35]
Calcium, iron, magnesium, cadmium, copper, nickel, lead, zinc, mercury, chrome	Atomic emission spectrometer (inductively coupled plasma with optical emission spectrophotometry)	3120B	[35]

**Table 4:** Initial microbiological properties of treated sewage sludge, soils and irrigation water.

Bacterium	S_sludge_ (CFU g^−1^)	S_0-clay_ (CFU g^−1^)	S_0-sand_ (CFU g^−1^)	S_w_ (CFU 100 mL^−1^)
Total Coliforms	2.5 ± 0.3 × 10^6^	1.3 ± 0.5 × 10^6^	8.4 ± 0.4 × 10^5^	1.3 ± 0.5 × 10^3^
*Escherichia coli*	6.3 ± 0.4 × 10^5^	2.0 ± 1.1 × 10^3^	4.8 ± 0.6 × 10^4^	<5.0 × 10^1^
*Staphylococcus aureus*	6.3 ± 0.9 × 10^5^	2.1 ± 0.9 × 10^4^	5.7 ± 0.3 × 10^4^	1.5 ± 0.8 × 10^2^
*Enterococcus* sp.	5.4 ± 0.5 × 10^6^	1.3 ± 1.2 × 10^3^	1.1 ± 0.7 × 10^2^	<1.0 × 10^1^
*Pseudomonas* sp.	5.6 ± 2.1 × 10^5^	1.1 ± 0.6 × 10^2^	2.6 ± 0.1 × 10^3^	1.8 ± 0.8 × 10^3^
Total Mesophylls	2.4 ± 0.3 × 10^7^	4.1 ± 0.2 × 10^7^	3.2 ± 0.4 × 10^7^	2.1 ± 0.3 × 10^3^
*Salmonella* sp.	Absence	Absence	Absence	Absence

**Table 5:** Initial physical-chemical properties of treated sewage sludge, soils and irrigation water.

Parameter	Units	S_sludge_	S_0-clay_	S_0-sand_	S_w_
pH	-	6.6 ± 0.1	7.1 ± 0.1	7.1 ± 0.1	7.2 ± 0.1
Temperature	ºC	16.0 ± 0.1	18.0 ± 0.2	18.0 ± 0.2	11.8 ± 0.1
Conductivity	μS cm^−1^	1105 ± 10	4200 ± 8	10715 ± 12	162 ± 5
Total Organic Carbon	mg g^−1^	480 ± 30	11 ± 1	14 ± 2	8 ± 1 (mg L^−1^)
Suspended Solids	mg L^−1^	-	-	-	110 ± 2
Total Solids	g g^−1^	0.25 ± 0.01	0.85 ± 0.01	0.87 ± 0.01	-
Organic Nitrogen	%	21.34 ± 0.05	0.86 ± 0.02	0.68 ± 0.01	-
Assimilable Phosphorous	mg kg^−1^	13.3 ± 0.2	9.8 ± 0.1	10.5 ± 0.2	-
Assimilable Potassium	mg kg^−1^	292 ± 21	200 ± 17	118 ± 14	-
Calcium	mg kg^−1^	49.39 ± 2.54	152.63 ± 3.59	161.30 ± 4.12	-
Iron	mg kg^−1^	8.04 ± 0.79	22.15 ± 1.57	17.58 ± 0.98	-
Magnesium	mg kg^−1^	3.78 ± 0.09	7.39 ± 0.15	4.34 ± 0.85	-
Cadmium	mg kg^−1^	<DL ^1^	<DL ^1^	<DL ^1^	-
Copper	mg kg^−1^	0.15 ± 0.01	<DL ^1^	<DL ^1^	-
Nickel	mg kg^−1^	0.04 ± 0.01	0.03 ± 0.01	0.03 ± 0.01	-
Lead	mg kg^−1^	<DL ^1^	<DL ^1^	<DL ^1^	-
Zinc	mg kg^−1^	0.69 ± 0.01	0.05 ± 0.01	0.05 ± 0.01	-
Mercury	mg kg^−1^	<DL ^1^	<DL ^1^	<DL ^1^	-
Chrome	mg kg^−1^	<DL ^1^	<DL ^1^	<DL ^1^	-

^1^ DL: detection limit.

**Table 6:** Evolution of physical and chemical parameters of clayey soil amended with sewage sludge.

Parameter	Units	S_0-clay_	S_i-clay_	S_3w-clay_	S_5w-clay_	S_42w-clay_
pH	-	7.1 ± 0.1	7.1 ± 0.1	7.0 ± 0.1	6.8 ± 0.1	7.2 ± 0.1
Temperature	ºC	18.0 ± 0.2	18.0 ± 0.1	13.0 ± 0.1	18.0 ± 0.1	10.4 ± 0.1
Conductivity	μS cm^−1^	4200 ± 8	4300 ± 15	9000 ± 10	9800 ± 10	11,000 ± 8
Total Organic Carbon	mg g^−1^	11 ± 1	25 ± 3	24 ± 2	22 ± 2	18 ± 2
Total Solids	g g^−1^	0.85 ± 0.01	0.85 ± 0.01	0.87 ± 0.01	0.84 ± 0.01	0.84 ± 0.01
Organic Nitrogen	%	0.86 ± 0.02	1.47 ± 0.07	0.87 ± 0.05	0.80 ± 0.06	0.78 ± 0.04
Assimilable Phosphorous	mg kg^−1^	9.8 ± 0.1	9.9 ± 0.2	8.5 ± 0.1	22.1 ± 0.3	17.1 ± 0.2
Assimilable Potassium	mg kg^−1^	200 ± 17	202 ± 19	214 ± 20	210 ± 18	212 ± 18
Calcium	mg kg^−1^	152.63 ± 3.59	149.53 ± 2.58	141.67 ± 3.01	134.89 ± 2.72	129.85 ± 1.54
Iron	mg kg^−1^	22.15 ± 1.57	21.73 ± 1.24	21.60 ± 1.24	21.46 ± 1.54	21.42 ± 1.57
Magnesium	mg kg^−1^	7.39 ± 0.15	7.28 ± 0.65	7.30 ± 0.25	7.43 ± 0.74	7.35 ± 0.74
Cadmium	mg kg^−1^	<DL ^1^	<DL ^1^	<DL ^1^	<DL ^1^	<DL ^1^
Copper	mg kg^−1^	<DL ^1^	<DL ^1^	<DL ^1^	<DL ^1^	<DL ^1^
Nickel	mg kg^−1^	0.03 ± 0.01	0.03 ± 0.01	0.03 ± 0.01	0.03 ± 0.01	0.03 ± 0.01
Lead	mg kg^−1^	<DL ^1^	<DL ^1^	<DL ^1^	<DL ^1^	<DL ^1^
Zinc	mg kg^−1^	0.05 ± 0.01	0.07 ± 0.01	0.06 ± 0.01	0.05 ± 0.01	0.05 ± 0.01
Mercury	mg kg^−1^	<DL ^1^	<DL ^1^	<DL ^1^	<DL ^1^	<DL ^1^
Chrome	mg kg^−1^	<DL ^1^	<DL ^1^	<DL ^1^	<DL ^1^	<DL ^1^

^1^ DL: detection limit.

**Table 7:** Evolution of physical and chemical parameters of sandy soil amended with sewage sludge.

Parameter	Units	S_0-sand_	S_i-sand_	S_3w-sand_	S_5w-sand_	S_24w-sand_
pH	-	7.1 ± 0.1	7.1 ± 0.1	7.3 ± 0.1	7.2 ± 0.1	7.1 ± 0.1
Temperature	ºC	18.0 ± 0.2	18.0 ± 0.1	13.2 ± 0.1	18.4 ± 0.1	16.2 ± 0.1
Conductivity	μS cm^−1^	10715 ± 12	10700 ± 12	11300 ± 10	10900 ± 8	11500 ± 11
Total Organic Carbon	mg g^−1^	14 ± 2	27 ± 2	26 ± 2	25 ± 1	27 ± 1
Total Solids	g g^−1^	0.87 ± 0.01	0.87 ± 0.01	0.89 ± 0.01	0.84 ± 0.01	0.92 ± 0.01
Organic Nitrogen	%	0.68 ± 0.01	1.3 ± 0.04	0.68 ± 0.05	0.60 ± 0.03	0.58 ± 0.03
Assimilable Phosphorous	mg kg^−1^	10.5 ± 0.2	10.6 ± 0.2	6.5 ± 0.1	20.5 ± 0.3	14.7 ± 0.2
Assimilable Potassium	mg kg^−1^	118 ± 14	122 ± 11	130 ± 12	136 ± 11	141 ± 13
Calcium	mg kg^−1^	161.30 ± 4.12	157.92 ± 3.48	140.57 ± 2.87	124.43 ± 1.95	127.56 ± 1.57
Iron	mg kg^−1^	17.58 ± 0.98	17.30 ± 0.57	17.21 ± 0.87	16.99 ± 0.15	15.17 ± 0.87
Magnesium	mg kg^−1^	4.34 ± 0.85	4.32 ± 0.45	4.39 ± 0.74	4.57 ± 0.65	4.39 ± 0.32
Cadmium	mg kg^−1^	<DL ^1^	<DL ^1^	<DL ^1^	<DL ^1^	<DL ^1^
Copper	mg kg^−1^	<DL ^1^	<DL ^1^	<DL ^1^	<DL ^1^	<DL ^1^
Nickel	mg kg^−1^	0.03 ± 0.01	0.03 ± 0.01	0.03 ± 0.01	0.03 ± 0.01	0.03 ± 0.01
Lead	mg kg^−1^	<DL ^1^	<DL ^1^	<DL ^1^	<DL ^1^	<DL ^1^
Zinc	mg kg^−1^	0.05 ± 0.01	0.07 ± 0.01	0.06 ± 0.01	0.05 ± 0.01	0.05 ± 0.01
Mercury	mg kg^−1^	<DL ^1^	<DL ^1^	<DL ^1^	<DL ^1^	<DL ^1^
Chrome	mg kg^−1^	<DL ^1^	<DL ^1^	<DL ^1^	<DL ^1^	<DL ^1^

^1^ DL: detection limit.
